# ^18^F-fluoride PET: changes in uptake as a method to assess response in bone metastases from castrate-resistant prostate cancer patients treated with ^223^Ra-chloride (Alpharadin)

**DOI:** 10.1186/2191-219X-1-4

**Published:** 2011-06-07

**Authors:** Gary JR Cook, Chris Parker, Sue Chua, Bernadette Johnson, Anne-Kirsti Aksnes, Val J Lewington

**Affiliations:** 1 Department of Nuclear Medicine and PET, Royal Marsden Hospital, Sutton, UK; 2 Academic Urology Unit, Royal Marsden Hospital and Institute of Cancer Research, Sutton, UK; 3 Algeta ASA, Oslo, Norway

## Abstract

**Background:**

A qualitative assessment of conventional bone scintigraphy with ^99m^Tc methylene diphosphonate is perceived as an insensitive method for monitoring the treatment response of bone metastases, and we postulated that semi-quantitative ^18^F-fluoride positron emission tomography (PET) might serve as a suitable alternative biomarker of the treatment response.

**Methods:**

Five patients with castrate-resistant prostate cancer and bone metastases with no known soft tissue disease received 100 kBq/kg of radium-223 (^223^Ra)-chloride (Alpharadin) therapy at 0 and 6 weeks and had whole body ^18^F-fluoride PET scans at baseline, 6 and 12 weeks with concurrent prostatic-specific antigen (PSA) and alkaline phosphatase (ALP) measurements. A qualitative comparison of the PET scans was performed blinded to the PSA and ALP results. A semi-quantitative comparison was made by measuring the maximum standardised uptake values (SUVmax) in five bone metastases in each patient. The means of the five SUVmax measurements in each subject were used as a quantitative measure of global metastatic activity at each time point.

**Results:**

Three patients showed a PSA decline at 12 weeks (-44%, -31%, -27% reduction) whilst two patients showed PSA increases (+10%, +17%). All five patients showed a reduction in ALP of greater than 25%. The qualitative assessment of the ^18^F-fluoride scans recorded a stable disease in each case. However, the semi-quantitative assessment showed agreement with the PSA decline in three patients (-52%, -75%, -49%) and minimal change (+12%, -16%) in two patients with increased PSA at 12 weeks. Four patients showed similar reductions in mean SUVmax and ALP at 12 weeks.

**Conclusions:**

The semi-quantitative ^18^F-fluoride PET is more accurate than the qualitative comparison of scans in assessing response in bone metastases, correlating with the PSA response and ALP activity and offering a potential imaging biomarker for monitoring treatment response in bone metastases following treatment with ^223^Ra-chloride.

## Background

Prostate cancer is the commonest cancer in men in the UK and is the second most common male cancer worldwide [1]. Bone metastases are common in patients with prostate cancer, and approximately 70% of patients have evidence of skeletal disease at post-mortem [2]. Bone metastases are associated with significant morbidity including pain, pathological fracture and cord compression, and the median survival is 20 months [2]. The demands on health care resources can be great, and it is therefore important that accurate methods are available to monitor therapy which can give an indication of success or failure early in the course of treatment as part of routine clinical management or within the context of clinical trials.

However, bone metastases are notoriously difficult to monitor during treatment, and in practice a combination of clinical, biochemical (e.g. prostate-specific antigen (PSA), serum alkaline phopshatase (ALP)) and imaging assessments are used [3]. However, it is generally accepted that the current methods are insensitive and often non-specific compared to those used for soft tissue disease. The measurement of response in bone metastases remains a challenge in routine oncological practice and in clinical trials, to the extent that bone metastases are often regarded as a non-measurable disease [4]. Criteria exist which use radiographic changes to measure response in bone metastases, but these are relatively insensitive, taking a number of months for changes to occur [5]. In addition, the criterion of sclerosis of previously osteolytic metastases is not relevant for metastatic disease that is predominantly sclerotic at baseline, as can occur in a number of cancers and is particularly common in prostate cancer. As an alternative to morphological imaging methods there has previously been some interest in using functional bone-specific imaging methods such as ^99m^Tc-methylene diphosphonate (MDP) scintigraphy. However, it has been shown that the bone scan is limited in its sensitivity to measure treatment effect, with only 52% of responders showing scintigraphic improvement and 62% of non-responders showing scintigraphic deterioration at 6 to 8 months in one early study of patients with breast cancer [6]. In an attempt to increase sensitivity of response measurement, some authors have described various semi-quantitative means of following bone metastases with bone scintigraphy [7-10], but others have found no advantage over visual interpretation [11] and quantitative methods have not gained acceptance into routine practice.

^18^F-fluoride was first described as a bone imaging agent nearly 40 years ago [12], but it is only in recent years, with improvements in positron emission tomography (PET) imaging equipment and resultant high-quality images, that there has been renewed interest in this tracer. Bone-specific imaging with ^18^F-fluoride PET and PET/CT has shown increased diagnostic accuracy compared to ^99m^Tc-MDP planar and/or single-photon emission computed tomography (SPECT) imaging in prostate and other cancers [13-18]. PET also offers the inherent advantage of superior quantitative accuracy over planar or SPECT scintigraphy, and we postulated that semi-quantitative ^18^F-fluoride PET might allow an accurate and timely measurement of the treatment response in bone metastases from prostate cancer. A previous report describes changes in ^18^F-fluoride uptake measured with PET following bisphosphonate treatment of glucocorticoid-induced osteoporosis [19], but to our knowledge, this method has not been used to monitor the treatment effects in bone metastases. The purpose of this pilot study was to demonstrate the early treatment response to radium-223 (^223^Ra)-chloride (Alpharadin, Algeta ASA, Oslo, Norway), a bone-seeking alpha emitter with a half-life of 11.4 days, using semi-quantitative ^18^F-fluoride PET and to compare with changes in biochemical markers including PSA as a tumour marker and ALP as a bone formation marker.

## Methods

This imaging study was performed as a pilot substudy of an open-label phase 1 trial of Alpharadin in patients with bone metastases and castration-resistant prostate cancer. Repeated ^18^F-fluoride PET imaging, PSA and ALP assessments were performed to assess treatment response. Biochemical results were retrieved from an institutional electronic patient record corresponding to the timing of the PET scans. Approval for this study was obtained from a research ethics committee and the national Administration of Radioactive Substances Advisory Committee, and the patients provided written informed consent.

Five male subjects with osteoblastic bone metastases from castration-resistant prostate cancer, as determined by ^99m^Tc-MDP bone scintigraphy and with no evidence of nodal or visceral disease on CT scan, were included in this pilot study. The mean age was 63.2 years (range, 57 to 70 years).

The subjects received 100 kBq/kg of ^223^Ra-chloride intravenously at baseline and after 6 weeks. PSA, ALP and ^18^F-fluoride PET measurements were performed at baseline before the first administration, at 6 weeks just before the second administration and at 12 weeks to assess treatment response.

A whole body ^18^F-fluoride PET imaging was performed 1 h following injection of 250 MBq ^18^F-fluoride on a Gemini PET/CT scanner (Philips Medical Systems, Cleveland, OH, US).

Data were acquired for 3.5 min per bed position following a low-current (50 mAs) CT scan performed for attenuation correction and lesion localisation. Scans were assessed qualitatively by visual inspection for evidence of response at 6- and 12-week time points compared to the baseline scan and without knowledge of the PSA or ALP results. The images were analysed using the Hermes Gold 3 software (Hermes Medical Solutions, Stockholm, Sweden). For the semi-quantitative assessment, five representative bone metastases were chosen that were greater than 2 cm in diameter in each subject. Lesions were selected to represent both axial and appendicular sites when present and the maximum standardised uptake value (SUVmax) calculated from an ROI of each lesion. The average SUVmax of the five lesions was then calculated for each patient at each scanning time point.

## Results

**Figure 1:**
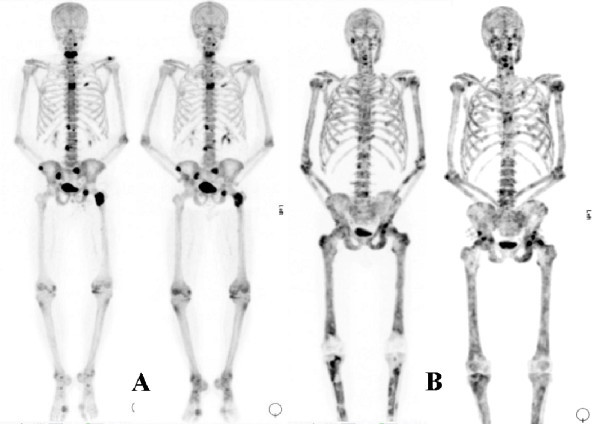
**Qualitative response assessment**. Maximum intensity projection images at 0 and 12 weeks in two subjects, subject A and subject B, showing no significant qualitative change.

**Figure 2:**
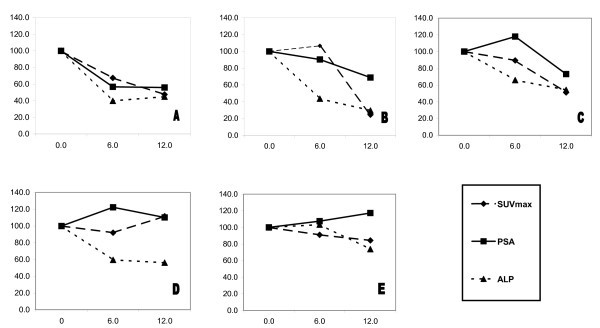
**Mean SUVmax, PSA and ALP changes**. Mean SUVmax, PSA and ALP changes at 6 and 12 weeks as a percentage of baseline levels in the five subjects (A to E).

The qualitative interpretation resulted in a classification of stable diseases in all five cases with no perceptible difference in uptake or number of lesions (Figure 1). In the semi-quantitative analysis, only one subject showed a significant reduction in mean SUVmax at 6 weeks (-32.5%, subject A in Figure 2), and this subject and two others showed a significant reduction at 12 weeks (-52.4%, -75.3% and -48.8%, subjects A, B and C, respectively in Figure 2) (Table 1). Two subjects showed minimal changes at 6 and 12 weeks (-8%, -9% at 6 weeks and +11.6%, -15.7% at 12 weeks, subjects D and E, respectively in Figure 2). The three subjects who showed reductions in mean SUVmax at 12 weeks also showed reductions in PSA and ALP. The two subjects that showed only minimal changes in mean SUVmax both showed a small rise in PSA (+10.2, +17.3%, respectively) but a greater than 25% drop in ALP at 12 weeks (Figure 2).

**Table 1:** Disease extent, measured metastatic sites and changes in mean SUVmax, PSA and ALP at 6 and 12 weeks after first administration of ^223^Ra

Subject (disease extent)	0 weeks				6 weeks			12 weeks		
	Measured sites	Mean SUVmax (range)	Baseline PSA (ng/ml)	Baseline ALP (U/L)	SUV (range) [% of baseline]	PSA [% of baseline]	ALP [% of baseline]	SUV (range) [% of baseline]	PSA [% of baseline]	ALP [% of baseline]
A (6-20 metastases)	C6, T6, L Sacrum, R ilium, L femur	46.4 (33.1-75.3)	370	118	31.3 (26.5-40.5) [67.5%]	210 [56.8%]	47 [39.8%]	22.1 (16.7-28.2) [47.6%]	207 [55.9%]	53 [44.9%]
B (superscan)	C3, L1, L5, L femur, R tibia	15.0 (13.2-17.2)	508	761	16.0 (11.9-21.1) [106.7%]	459 [90.4%]	332 [43.6%]	3.7 (2.9-4.8) [24.7%]	350 [68.9%]	225 [29.6%]
C (6-20 metastases)	L1, L3, L5, sternum, R ischium	74.6 (54.7-98)	78	129	66.7 (51.3-76.7) [89.4%]	92 [117.9%]	85 [65.9%]	38.2 (26.5-46.9) [51.2%]	57 [73.1%]	70 [54.3%]
D (>20 metastases)	Skull, L scapula, T11, L3, L ilium	27.5 (20.3-35.4)	551	89	25.3 (20.6-29.5) [92%]	674 [122.3%]	53 [59.6%]	30.7 (21.1-59.4) [111.6%]	607 [110.2%]	50 [56.2%]
E (superscan)	Skull, T12, L3, R ilium, R femur	22.3 (11.4-28.6)	254	393	20.3 (11-25) [91%]	273 [107.5%]	406 [103.3%]	18.8 (9.8-24.8) [84.3%]	298 [117.3%]	290 [73.8%]

## Discussion

This pilot study has shown the feasibility of measuring changes in the uptake of ^18^F-fluoride with PET in patients with bone metastases from prostate cancer receiving systemic therapy with ^223^Ra-chloride. Additionally, it has shown that the semi-quantitative analysis using SUVs is able to measure changes not detected by qualitative visual inspection.

In this study, three subjects (A, B and C) showed a comparable reduction in ^18^F-fluoride uptake in bone metastases and in the biochemical parameters, PSA and ALP at 12 weeks. A more consistent correlation was present at 12 weeks than at 6 weeks. In two other subjects (D and E), small changes in ^18^F-fluoride uptake of less than 20% were seen, accompanied by small rises in PSA of less than 20% in both subjects but with larger changes in ALP (-43.8% and -26.2%, respectively) at 12 weeks.

^18^F-fluoride is a bone-specific tracer and rather than indicating tumour metabolic activity directly, it is a marker of the local osteoblastic reaction of bone that accompanies most bone metastases [20]. In accordance with this, a close correlation was seen between the magnitude of reduction in ^18^F-fluoride uptake and ALP at 12 weeks in four out of five of the subjects (Figure 2).

A potential weakness of an indirect assessment of tumour activity by measuring parameters that primarily reflect osteoblastic activity (i.e. ALP and ^18^F-fluoride uptake) is a temporal discordance in changes with intrinsic tumour activity. Although not observed in this pilot study, the osteoblastic reaction to a metastasis by bone may subside at a different rate than the metabolic activity of the malignant tumour tissue, or an initial increase in osteoblastic activity may occur as part of a healing response in the surrounding bone of responding metastases, the so-called flare phenomenon [21]. However, in this study, although not directly measuring tumour cell activity, as might be possible with other PET tracers including ^18^F-fluorodeoxyglucose or radiolabelled choline, changes in ^18^F-fluoride uptake were similar to changes in PSA at 12 weeks.

Our study suggests that a 12-week semi-quantitative assessment of ^18^F-fluoride uptake is a closer predictor of the biochemical PSA response than at 6 weeks. Previous reports of the flare phenomenon measured biochemically with ALP [22] or with quantitative bone scintigraphy [8] show that it peaks and substantially subsides before 2 months. These data may explain in part why the 12-week ^18^F-fluoride PET scan is a more reliable measure of tumour response than the 6-week time point. An assessment at 12 weeks after commencing systemic therapy for bone metastases remains at an early enough time point to be clinically relevant in informing clinical management decisions and for measuring early response in clinical trials.

A qualitative visual assessment of response using ^18^F-fluoride PET was insensitive to changes at 6 or 12 weeks in this study, and it is possible that for the same reason, a qualitative interpretation of the ^99m^Tc-MDP bone scintigraphy has not found universal favour and is relatively insensitive as a response assessment method, unless there are unequivocal new lesions beyond the expected flare period. In the absence of better imaging biomarkers, PET also has the inherent advantage over the conventional ^99m^Tc-MDP bone scintigraphy of more accurate and absolute quantification of radioactive tracer concentrations, and therefore lends itself to a quantitative approach. A potential weakness of the methodology employed in this study was that the semi-quantitative assessment was only performed in five lesions in each subject. However, the lesions were chosen to represent different areas of the skeleton and whilst a global measure of skeletal metastases would be of utility, this has practical limitations, particularly in patients with extensive disease. A full kinetic analysis of the dynamic PET and blood data would also be of interest, but this approach, whilst potentially more sensitive to small changes in bone clearance of tracer, is also limited in practicality.

These results are from a small pilot study, and the conclusions on the ability of ^18^F-fluoride PET to measure early response in bone metastases are limited. In addition, it is not possible to extrapolate these results to different forms of systemic therapy, including hormones or chemotherapy, or in different tumour types where the mechanism of action of individual drugs and the differences in biological behaviour of tumour types and effects on the skeleton may differ. Nevertheless, we believe that the results are of sufficient interest to encourage further research into using ^18^F-fluoride PET as a biomarker of response in bone metastases in a variety of cancers and treatment types.

## Conclusions

The results of this pilot study suggest there may be a role for semi-quantitative ^18^F-fluoride PET as a method to monitor the treatment response in bone metastases following systemic therapy with ^223^Ra-chloride at clinically relevant intervals.

## Abbreviations

ALP: alkaline phosphatase; MDP: methylene diphosphonate; PET: positron emission tomography; PSA: prostate-specific antigen; SUVmax: maximum standardised uptake value.

## Competing interests

CP is a consultant to Algeta ASA.

## Authors' contributions

GC study design, data acquisition, analysis, manuscript drafting and approval. CP study design, coordination of study, manuscript drafting and approval. SC data acquisition, manuscript drafting and approval. BJ coordination of study, patient recruitment, manuscript drafting and approval. AKA study design, coordination of study, manuscript drafting and approval. VL study design, data acquisition, analysis, manuscript drafting and approval.
